# Evaluating the necessity of PCR duplicate removal from next-generation sequencing data and a comparison of approaches

**DOI:** 10.1186/s12859-016-1097-3

**Published:** 2016-07-25

**Authors:** Mark T. W. Ebbert, Mark E. Wadsworth, Lyndsay A. Staley, Kaitlyn L. Hoyt, Brandon Pickett, Justin Miller, John Duce, for the Alzheimer’s Disease Neuroimaging Initiative, John S. K. Kauwe, Perry G. Ridge

**Affiliations:** Department of Biology, Brigham Young University, Provo, UT USA

**Keywords:** Next-Generation Sequencing, PCR duplicate removal, SAMTools, Picard

## Abstract

**Background:**

Analyzing next-generation sequencing data is difficult because datasets are large, second generation sequencing platforms have high error rates, and because each position in the target genome (exome, transcriptome, etc.) is sequenced multiple times. Given these challenges, numerous bioinformatic algorithms have been developed to analyze these data. These algorithms aim to find an appropriate balance between data loss, errors, analysis time, and memory footprint. Typical analysis pipelines require multiple steps. If one or more of these steps is unnecessary, it would significantly decrease compute time and data manipulation to remove the step. One step in many pipelines is PCR duplicate removal, where PCR duplicates arise from multiple PCR products from the same template molecule binding on the flowcell. These are often removed because there is concern they can lead to false positive variant calls. Picard (MarkDuplicates) and SAMTools (rmdup) are the two main softwares used for PCR duplicate removal.

**Results:**

Approximately 92 % of the 17+ million variants called were called whether we removed duplicates with Picard or SAMTools, or left the PCR duplicates in the dataset. There were no significant differences between the unique variant sets when comparing the transition/transversion ratios (*p* = 1.0), percentage of novel variants (*p* = 0.99), average population frequencies (*p* = 0.99), and the percentage of protein-changing variants (*p* = 1.0). Results were similar for variants in the American College of Medical Genetics genes. Genotype concordance between NGS and SNP chips was above 99 % for all genotype groups (e.g., homozygous reference).

**Conclusions:**

Our results suggest that PCR duplicate removal has minimal effect on the accuracy of subsequent variant calls.

## Background

Next-generation sequencing (NGS) has accelerated research efforts in virtually every field in the life sciences. NGS is being used to diagnose and determine the genetic cause of diseases, measure gene expression, refine phylogenetic trees, identify markers to differentiate between morphologically similar species, and *de novo* sequencing for non-model organisms [1–5]. For many years these types of projects were not possible because the data were difficult and expensive to obtain. Today, however, it is possible to sequence entire genomes for a fraction of what it cost just 10 years ago.

Despite the many benefits of NGS, these data are challenging to work with for several reasons, including: (1) NGS has a much higher error rate than other genotyping methods (e.g. compared to Sanger sequencing), (2) the most common NGS methods only produce short fragments, known as “reads”, ranging from ~100-300 nucleotides in length, and (3) datasets are very large, frequently >100 gigabytes [6]. Many experimental and bioinformatics innovations are employed to address these challenges.

One innovation to overcome the high error rate is to sequence each nucleotide (position) in the target DNA (genome, exome, etc.) multiple times. The number of times each nucleotide is sequenced is referred to as coverage. Coverage is variable within a sample and typical coverage ranges from 30 or less to >1000 for typical human genetic and cancer applications, respectively. This approach is employed under the assumption that sequencing errors are random, making deeper coverage more reliable to determine the nucleotide at a given position. In other words, if each nucleotide is sequenced multiple times, most reads will have the correct nucleotide. PCR duplicates are, at least in theory, one possible impediment to this innovation.

To prepare DNA for NGS, DNA is sonicated, and adapters are ligated to the end of each resulting fragment. Fragments are then PCR amplified and PCR products are spread across the flowcell. There are several additional steps not pertinent to this research, but have been previously described thoroughly by Voelkerding et al. [7]. PCR duplicates are sequence reads that result from sequencing two or more copies of the exact same DNA fragment, which, at worst, may contain erroneous mutations introduced during PCR amplification, or, at the very least, make the occurrence of the allele(s) sequenced in duplicates appear proportionately more often than it should compared to the other allele (assuming a non-haploid organism). Ideally, one PCR copy of each original DNA fragment will hybridize to the flowcell, but there is currently no way to enforce this. When multiple copies originating from the same DNA molecule all bind to the flowcell, each is sequenced and the resulting reads are referred to as PCR duplicates. These duplicates occur for two reasons: (1) we cannot control exactly which sequences from the pool of PCR products hybridize to the flowcell, and (2) not all of the original DNA molecules are amplified without bias (PCR amplification bias). PCR amplification bias and increasing the number of PCR cycles both increase the likelihood of PCR duplicates during sequencing.

Many analysis pipelines remove PCR duplicates to mitigate potential biases on variant calling algorithms. For example, a large number of PCR duplicates containing an amplification-induced error may cause a variant calling algorithm to misidentify the error as a true variant. Several programs exist to remove or mark PCR duplicates (e.g. SEAL [8], elPrep [9], FastUniq [10], etc.), but in this work we focus on the two most commonly used approaches: Picard MarkDuplicates (http://broadinstitute.github.io/picard/) and SAMTools rmdup [11, 12].

SAMTools and Picard use similar approaches for duplicate marking or removal, but with some differences. SAMTools (rmdup) identifies PCR duplicates by identifying pairs of reads where multiple reads align to the same exact start position in the genome, and the reverse read on the 3′ end maps at the exact same location (i.e. external mapping coordinates are identical). The read pair with the highest mapping quality score is retained and other read pairs removed (a possible disadvantage because data is lost). Also, rmdup does not work for unpaired reads (in paired end mode) or read pairs where each read maps to different chromosomes. There will also be unexpected results if multiple libraries are present in the same BAM file since rmdup assumes all reads in the BAM file originated from the same library [11, 12]. Picard (MarkDuplicates) is similar to rmdup. MarkDuplicates identifies read pairs with the same orientation that have the exact same 5′ start position in the mapping. It takes into account clipping on the 5′ end of the read and makes calculations based on where the 5′ start position would be if the entire read had mapped to the reference. In contrast to rmdup, MarkDuplicates handles interchromosomal read pairs, and considers the library for each read pair and keeps a read pair from each library. MarkDuplicates also does not remove reads, but sets the SAM flag 1024 for all but the best read pair. The best read pair is the read pair with the highest sum of base qualities with Q ≥ 15 (http://broadinstitute.github.io/picard/).

We performed a three-way comparison between variant calls generated without removing duplicates and those removing duplicates with either Picard MarkDuplicates or SAMTools rmdup to determine: (1) if PCR duplicate removal improves the accuracy of variant calls, and (2) if so, whether MarkDuplicates or rmdup produces a more accurate variant dataset. Our results suggest that accuracy is the same for both MarkDuplicates and rmdup, but there are substantial performance (execution time and memory usage) differences between the two. Our results further suggest that removing duplicates may not be necessary in variant calling pipelines.

## Methods

### Dataset

Whole-genome (WGS) data used in this article were obtained from the Alzheimer’s Disease Neuroimaging Initiative (ADNI) database (adni.loni.usc.edu). ADNI is the result of efforts of many co-investigators, led by Dr. Michael Weiner, from a broad range of institutions, and includes subjects from more than 50 American and Canadian research sites. A primary goal of ADNI is to identify biological markers for Alzheimer’s disease (AD). To date over 1,500 adults, ages 55 to 90, have participated in the study. For up-to-date information see www.adni-info.org. Of the 809 WGS samples (average coverage ~37) available in this dataset, we randomly selected 100 to use in our duplicate removal analysis. During the analysis process, one sample was removed due to low quality data and was not replaced. Each of the remaining 99 study samples was run through the exact same pipeline described below (Data Analysis subsection).

We also have matching SNP chip data for the 99 samples used in this study. Samples were genotyped using the HumanOmniExpress BeadChip Kit by Illumina. The SNP chip data were cleaned by removing (in order): (1) all SNPs missing greater than 2 % of data, (2) all individuals missing more than 2 % of data, (3) SNPs with a minor allele frequency less than 0.02, and (4) SNPs out of Hardy-Weinberg equilbrium (*p* < 0.000001).

### Data analysis

**Fig. 1:**
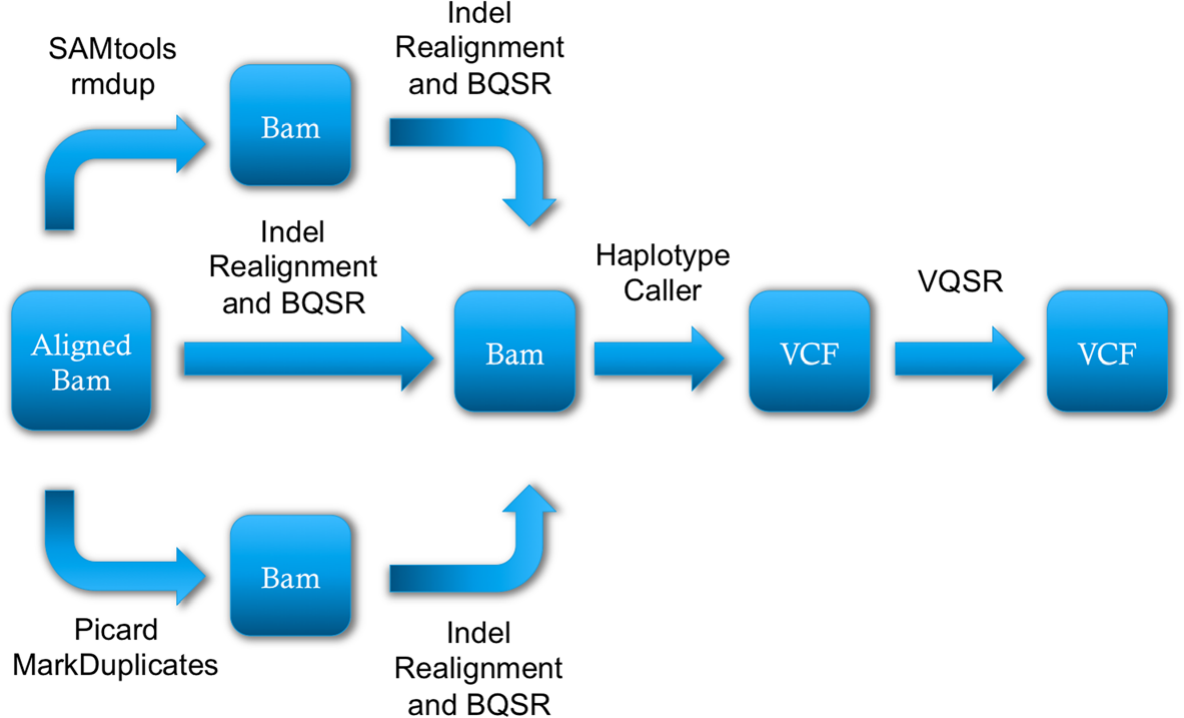
Our pipeline was identical for every sample, except for how we handled PCR duplicates. Original BAM files with mapped reads were processed using the shown pipeline. PCR duplicates were ignored, removed using SAMTools (rmdup), or marked (left in the file, but ignored in subsequent steps) using Picard (MarkDuplicates). After the duplicates step, all subsequent steps in the analysis pipeline were identical. The final output of the pipeline is a multi-sample VCF file. VCF: Variant Call Format, BQSR: Base Quality Score Recalibration, VQSR: Variant Quality Score Recalibration

We followed the GATK Best Practices [13] during this process, varying only the step of how and whether we removed duplicates during the process (Fig. 1). Genomes used in this research were aligned by ADNI using the Burrows-Wheeler Aligner (BWA) [14]. Three versions of each BAM file in our dataset were generated: (1) a BAM where PCR duplicates were left intact, (2) a BAM where PCR duplicates were removed using SAMTools (rmdup), and (3) a BAM where PCR duplicates were marked (and subsequently ignored) using Picard (MarkDuplicates). Subsequent steps are identical in each pipeline and all steps were performed with the Genome Analysis Toolkit (GATK, version 3.2). Following duplicate removal (or not) we refined the mappings using GATK’s IndelRealigner and BaseRecalibrator (BQSR), and joint called/refined variants using the HaplotypeCaller and variant quality score recalibration (VQSR).

### Variant Call Format (VCF) file comparisons

After completing the variant calling step we compared the three resulting variant call format (VCF) [15] files using Variant Tool Chest (VTC) [16]. VTC performs complex set operations, like intersect and complement, on VCF files. Next, we extracted summary statistics for the resulting intersects and complements with the variant statistics tool (VarStats) in VTC. We used R (version 3.1.2) to analyze the information from the comparisons of the three files, and the summary statistics [17].

### Comparison between SNP chip and NGS data

We compared results between each group and the matching SNP chip data, using the more accurate SNP data as truth. We measured percentage of genotypes changed from one type to another (e.g. heterozygous to homozygous variant).

### The American College of Medical Genetics (ACMG) gene list

In 2013, the American College of Medical Genetics (ACMG) published guidelines for reporting incidental findings in large sequencing diagnostics [18], typically defined as whole exome or genome sequencing, or sequencing targeted genes. Included in these guidelines is a list of genes the working group recommends clinicians always examine for deleterious mutations. The list was compiled based on conditions that are verifiable using secondary diagnostic approaches, and for which early intervention is likely to significantly change or prevent disease. This list is certainly not a comprehensive listing of all clinically important genes, but it does include genes that meet the criteria outlined above. The genes on the recommended list are perhaps the most clinically important known genes because there are effective treatments for disorders resulting from mutations in these genes. We refer to this gene list as the ACMG genes.

### Variant annotation

We used ANNOVAR [19] to annotate each variant with dbSNP 138 [20] identification numbers (if present in dbSNP), 1000 Genomes Project minor allele population frequency (if observed in the 1000 Genomes Project) [21, 22], and separated variants by type (e.g. nonsynonymous, InDel, etc.). We refer to protein changing variants as nonsynonymous variants, InDel frameshifting variants, or structural variants. We assumed any variant not present in dbSNP is novel.

### Duplicate removal

We calculated the percentage of duplicates removed by both Picard MarkDuplicates and SAMTools rmdup to quantify approximately how many reads were considered duplicates by both softwares, by comparing the number of reads in the BAM files before and after duplicate marking/removal. For MarkDuplicates, specifically, we counted the number of reads not marked as duplicates.

## Results

### Whole genome variant dataset: Picard versus SAMTools versus not removing duplicates

We processed whole genome data for each of 99 different genomes three different times. For one set of the 99 genomes, we removed duplicates using Picard (MarkDuplicates), for another set we removed duplicates using SAMTools (rmdup), and for the third we left the duplicates in the alignments. Next, we called variants on each of the alignments using the GATK pipeline (outlined above). Finally, we pooled all of the variants for each of the three sets of genomes for comparison. From this point forward, variant datasets referred to as Picard, SAMTools, or no dup refer to the union of variants from all 99 genomes with duplicates removed using Picard, SAMTools, or no duplicates removed, respectively.

**Fig. 2:**
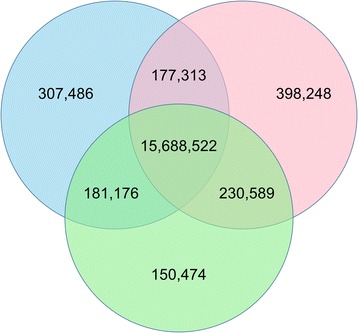
We constructed a Venn diagram using the variant datasets. The datasets correspond to the three pipelines: removing duplicates using SAMTools, removing duplicates using Picard, and ignoring duplicates. The blue circle is the Picard dataset, red is the no duplicates removed dataset, and green is the SAMTools dataset

In Fig. 2, we show the overlap of called variants in each of the datasets using a Venn diagram. There were a total of 17134081 different variants called, and about 16 million (about 92 %) were called regardless of how duplicates were treated (three-way intersect in the center of the Venn diagram in Fig. 2). Picard and no dup had about twice as many unique variants as SAMTools (307486 and 398248 compared to 150474), and the three two-way intersections each had comparable numbers of variants (177313, 181176, and 230589). Approximately 31 % of all variants in this study were rare, with a minor allele frequency less than 0.01. This is unsurprising because the 1000 Genomes Project demonstrated that each individual has hundreds of rare variants at evolutionarily conserved sites alone [22].

Next we analyzed the variant characteristics from individual partitions of the Venn diagram (Table 1). Several measurements can be used to assess the quality of a variant dataset, such as total number of variants, transition/transversion (Ti/Tv) ratios, proportion of novel variants, and proportion of variants that change the protein product. The number of SNPs between sets (labeled “All” in Table 1) and subsets (labeled “Unique” in Table 1) were significantly different, but the Ti/Tv ratios, percentage of variants in dbSNP, population frequencies, and percentage of variants that are protein changing are not significantly different (Table 1). Variants across the full Picard, SAMTools, and no dup sets had about 16 million total variants, Ti/Tv ratios of 2.14, 28–29 % novel variants, and 0.4 % of variants are protein-changing. Variants across the subsets (labeled “Unique” in Table 1) had lower Ti/Tv ratios and percentage of variants changing the protein, and higher novel variants.

**Table 1:** Minimal differences between Picard, SAMTools, and no duplicate removal

Subset	Total Variants	Ti/Tv Ratio	% Variants in dbSNP	Avg. Population Frequency	% Protein Changing Variants
All Picard	16354497	2.14	72.05	0.21	0.40
All SAMTools	16250761	2.14	71.86	0.22	0.40
All No Dups	16494672	2.14	71.30	0.21	0.40
*P*-Value	<2.60e-16	1.00	0.99	0.99	1.00
Common to all three	15688522	2.15	80.18	0.22	0.41
Unique to Picard	307486	1.92	66.27	0.16	0.33
Unique to SAMTools	150474	1.80	69.59	0.19	0.26
Unique to No Dups	398248	1.95	54.07	0.16	0.34
Unique to Picard/SAMTools	181176	1.97	73.86	0.22	0.33
Unique to Picard/No Dups	177313	2.07	65.30	0.21	0.31
Unique to SAMTools/No Dups	230589	1.73	52.17	0.23	0.24
*P*-Value (comparing Unique rows)	<2.60e-16	1.00	0.32	0.84	1.00

Here we present metrics from each portion of the Venn diagram (Fig. 2), including total number of variants, transition/transversion (Ti/Tv) ratios, average population frequency, proportion of novel variants, and proportion of variants that change the protein product. In the top part of the table, variant characteristics are reported for all the variants resulting from duplicate removal using Picard or SAMTools, or no duplicate removal. Variants from the dataset processed using Picard are referred to as Picard, processed using SAMTools as SAMTools, and the dataset without duplicate removal as No Dups. Population frequencies are based on the 1000 Genomes Project, dbSNP variants refer to build 138 and any variant not present in dbSNP is considered novel, and protein changing variants are missense SNVs or frameshifting InDels. We performed a Chi-square goodness-of-fit to test for significant differences amongst values in each column. Two tests were performed for each column: (1) comparing the values for all variants in each main dataset (“All Picard”, “All SAMTools”, and “All No Dups”); and (2) comparing values for variants across all “Unique” groups. There was a significant difference when comparing the number of variants across groups, but none of the other measures were significantly different

To further compare these variant datasets, we compared the intersections of the three variant datasets. Most of the variants (15.6 million of ~17 million) were called using all three approaches. Metrics for each of the individual variant datasets and the three-way intersect were very comparable, except that ~10 % fewer variants were novel in the three-way intersect (Table 1). Variant characteristics in different partitions of the datasets (e.g. Unique to Picard/SAMTools, Unique to SAMTools/no dups, Unique to SAMTools, etc.) are dramatically different than the three-way intersect. The biggest changes occur in Ti/Tv ratios and percent novel variants. Except for the three-way intersect and entire variant datasets for each of the three approaches, the Ti/Tv ratios are all less than two, in contrast to ~2.1. Additionally, a large number of novel variants exist in each of these other partitions. There are more novel variants in the Unique to No Dups (~46 %) and Unique to SAMTools/No Dups (~48 %) groups.

### ACMG genes variant dataset: Picard versus SAMTools versus not removing duplicates

In 2008 the American College of Medical Genetics (ACMG) compiled a list of genes known to harbor disease-causing mutations (i.e. clinically important genes). We compared variants in only the ACMG genes to determine if the choice of duplicate removal appears to be (more or less) important for the study of clinically important genes.

**Fig. 3:**
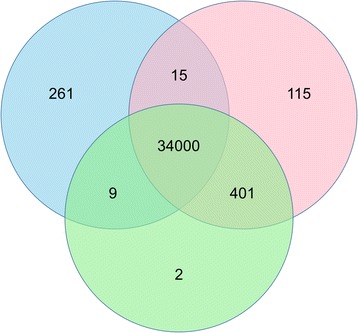
The Venn diagram is as described in Fig. 2, except limited to variants in the ACMG genes. The blue circle is the Picard dataset, red is the no duplicates removed dataset, and green is the SAMTools dataset

We performed the same partitioning of variants (Fig. 3) and comparison of variant characteristics (Table 2) as above. Many of the results were comparable. The majority (34000 of 34803, ~98 %) of variants were identified using all three approaches. Again, the variants in the three-way intersect were very similar compared to all the ACMG variants in the each of the three individual datasets. However, these variants (three-way intersect and whole ACMG variant datasets) and the other partitions of ACMG variants, were very different in every measured statistic.

**Table 2:** Differences using only ACMG genes are also minimal

Subset	Total Variants	Ti/Tv Ratio	% Variants in dbSNP	Avg. Population Frequency	% Protein Changing Variants
All Picard	34285	2.29	67.75	0.20	1.08
All SAMTools	34412	2.29	67.51	0.20	1.08
All No Dups	34531	2.29	67.34	0.20	1.07
*P*-Value	0.64	1.00	0.99	1.00	1.00
Common to all three	34000	2.31	76.37	0.20	1.09
Unique to Picard	261	1.01	19.64	0.88	0
Unique to SAMTools	2	1.00	0	NA	0
Unique to No Dups	115	1.54	22.33	0.04	0
Unique to Picard/SAMTools	9	1.50	40	0.03	0
Unique to Picard/No Dups	15	1.00	0	NA	0
Unique to SAMTools/No Dups	401	0.98	15.86	0.12	0.32
*P*-Value (comparing Unique rows)	<2.60e-16	0.998	1.04e-13	0.74	0.90

We performed the same analyses using only the ACMG genes and found similar results

### Comparison between SNP chip and NGS data

We grouped variants from the SNP chip by variant type (i.e. homozygous reference, heterozygous, or homozygous alternate) and compared each of these genotypes to the called genotypes in each of the three groups of NGS data. Comparing genotypes across the NGS and matching SNP chip data, we found that Picard, SAMTools, and not removing duplicates were virtually indistinguishable (Table 3). Exactly 99.97 % of genotypes called homozygous reference by SNP chips were also called homozygous reference by NGS across no dup, picard, and SAMTools. Similarly, 99.91 % of ACMG genotypes called homozygous reference by SNP chips were called identically by NGS. NGS genotypes were equally accurate in both whole genome and ACMG comparisons for the other two classes of genotypes (Table 3).

**Table 3:** Concordance between SNP chip and NGS data across all three duplicate removal methods are nearly identical

		Chip data		
		homref	het	homalt
	homref	99.97	0.18	0.16
No dup	het	0.01	99.81	0.13
	homalt	0.02	0.01	99.71
	homref	99.97	0.19	0.14
Picard	het	0.01	99.80	0.14
	homalt	0.02	0.01	99.71
	homref	99.97	0.19	0.16
SAMTools	het	0.01	99.80	0.13
	homalt	0.02	0.01	99.71
	homref	99.91	0.06	0.18
No dup ACMG	het	0.02	99.94	0.07
	homalt	0.08	0.00	99.76
	homref	99.91	0.06	0.18
Picard ACMG	het	0.02	99.94	0.08
	homalt	0.08	0.00	99.75
	homref	99.91	0.06	0.18
SAMTools ACMG	het	0.02	99.94	0.07
	homalt	0.08	0.00	99.76

We compared the genotypes from NGS and matched SNP chip data to see if concordance varied by duplicate removal approaches. We performed this comparison for all variants and for ACMG variants only. Reported values are the percentage of total SNP chip genotypes called for a particular group (e.g., homozygous reference) that were correctly called by NGS for a given group. Exactly 99.97 % of genotypes called homozygous reference by SNP chip were also called homozygous reference by NGS across no dup, picard, and SAMTools. Similarly, 99.91 % of ACMG genotypes called homozygous reference by SNP chip were called identically by NGS. In the Table, homref: homozygous for the reference allele, het: heterozygous, and homalt: homozygous for an alternate allele

### Computational performance: Picard versus SAMTools

**Fig. 4:**
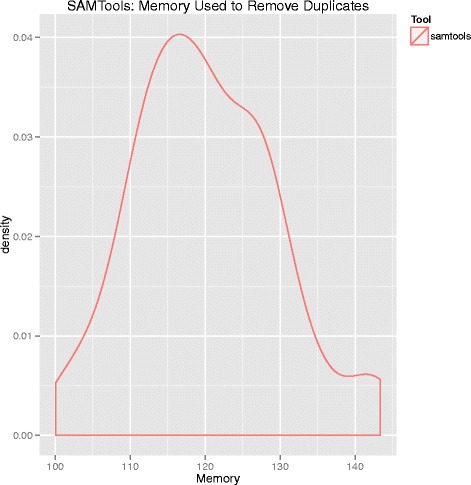
Density plot of memory usage by SAMTools for duplicate removal. The y-axis is memory in megabytes. Note the difference in magnitude of memory used in this figure compared to Picard (Fig. 5)

**Fig. 5:**
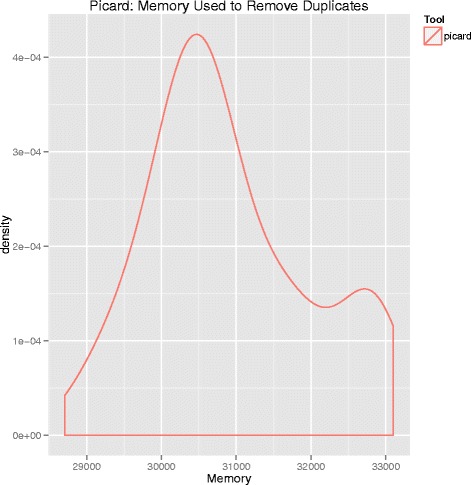
Density plot of memory usage by Picard for duplicate removal. The y-axis is memory in megabytes. Note the difference in magnitude of memory used in this figure compared to SAMTools (Fig. 4)

**Fig. 6:**
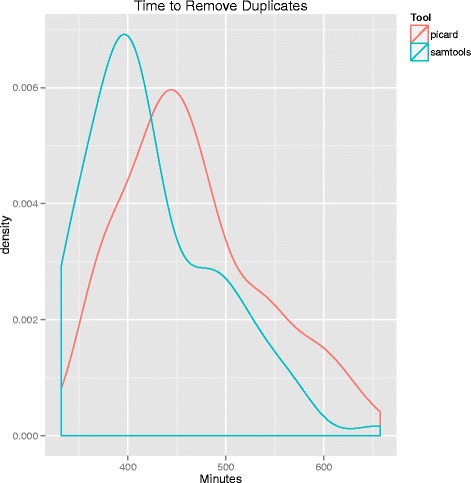
Density plot of execution time for both SAMTools and Picard duplicate removal. Picard is marked by the red line, and SAMTools in blue. The y-axis is execution time measured in minutes

We compared memory usage and compute time to assess the relative performances of Picard and SAMTools. Memory usage and compute time are summarized in Figs. 4, 5, and 6, respectively. Picard required both more memory and execution time than SAMTools. Picard used an average of 31000 megabytes of memory and had an average execution time of almost eight hours. In contrast, SAMTools on average used 120 megabytes of memory and had an average run time of about seven hours.

### Duplicate removal

The average number of reads across the 99 samples was 1313199168 with a range of 1051352190 to 1734787274. The number of duplicates identified by both softwares was comparable, though Picard removed more reads on average. The average percentage of reads marked by Picard was 1.8 % and the average removed by SAMTools was 1.1 %.

## Discussion

PCR duplicate removal is a recommended step in nearly every variant calling pipeline for NGS data. It is a both a memory and time intensive step, and results in varying percentages of reads being removed. There is no question about whether or not removed reads are valid, or real, sequence reads. Therefore, removing or ignoring PCR duplicates results in ignoring some of the generated sequence data. Two different algorithms are predominantly used for PCR duplicate removal (Picard and SAMTools). To our knowledge, no one has formally compared the two different algorithms. Furthermore, there is little data assessing the necessity of PCR duplicate removal. Our goal was to determine whether PCR duplicate removal meaningfully affects the resulting variant datasets, and if the accuracy of the variant datasets is different when using Picard and SAMTools.

We compared the variant datasets resulting from each of the three different pipelines. First, we compared common measures of the variants in each dataset to assess the overall quality of the called variants [23]. This comparison says nothing about any individual variant, but about the accuracy of the dataset as a whole. When considering the entire variant dataset for each of the three approaches, the important characteristics we compared were nearly identical. All three had Ti/Tv ratios of 2.14, a ratio in the expected range [12], very similar proportions of novel variants (27.95 % to 28.7 %) also in the expected range [23], identical minor allele frequencies for called variants (21 %), and identical proportions of protein changing variants (0.4 %). Therefore, using these meaningful measures to assess the accuracy of the variant calls, the three approaches are nearly indistinguishable. There is evidence suggesting that the intersection of the three variant datasets results in a more accurate dataset because the percentage of novel variants decreased by a small amount (~27 % to ~20 %), though the difference was not significant when comparing the average of the three unique values to the intersect of all three (*p* = 0.49). However, when looking at all other possible intersects of the three variant datasets, the apparent quality of the datasets drops.

We further assessed the accuracy of the three variant datasets by comparing genotypes in the variant datasets to genotypes from SNP chip data. All three approaches were much more accurate than 99 % and nearly identically accurate. This comparison, however, only demonstrates concordance amongst common variants. In this case, we only considered variants with a minor allele frequency greater than 0.02.

As NGS is being moved into a clinical setting, we wanted to verify that our results are consistent in clinically important genes so we performed the same tests in parallel in only the ACMG genes. The ACMG recommended a set of genes that should be assessed when analyzing the whole genome/exome of a patient in a clinical setting. We make no assumption that this gene set contains every clinically important gene, but it does contain all the genes considered most important by the ACMG. We performed the same analyses on variants from only the genes recommended by the ACMG, and our findings are nearly identical to those performed on variants from the entire genome. The three individual datasets are indistinguishable, while the intersection of the three again appears to be slightly better, and there is still nearly perfect concordance with SNP chip data.

Next, to assess computational performance differences between Picard and SAMTools, we measured the memory and compute time required for each to remove duplicates. Picard required substantially more memory (31000 versus 120 megabytes) and slightly more time (seven versus eight hours) than SAMTools.

Removing duplicates is intended to reduce noise during the variant identification process and minimize false positives. Our results suggest removing duplicates has little effect on the results. As sequencing technologies continue to advance, PCR duplicate removal will become less of an issue. For example, single-molecule sequencing technologies such as PacBio’s Single Molecule, Real-Time (SMRT) sequencing and Oxford Nanopore Technologies’ Minion perform sequencing on non-amplified DNA.

## Conclusions

In summary, we compared the effect on the resulting variant datasets when using Picard for duplicate removal, SAMTools for duplicate removal, or not removing duplicates. We performed these comparisons across the entire genome, and then limited our analyses to variants located in clinically important genes. Our results suggest that in deep sequencing whole genome data, removing or ignoring PCR duplicates has non-significant effects on the accuracy of subsequent variant datasets. Furthermore, our results demonstrate that when PCR duplicates are handled using either SAMTools or Picard, the resulting variant datasets are very comparable. In some settings, PCR duplicate removal/marking may be preferable. For example, our data show that the most accurate variant dataset may be obtained by using each of the three approaches and then intersecting the three datasets (assuming any variant outside the intersect is a false positive). Since NGS library preparation is different for sequencing genomes, exomes, transcriptomes, etc. additional studies will be necessary to know if our results extend to the exome or other sequenced partitions of the genome.
